# Immunodominant antigens that induce Th1 and Th17 responses protect mice against *Helicobacter pylori* infection

**DOI:** 10.18632/oncotarget.23927

**Published:** 2018-01-03

**Authors:** Heqiang Sun, Hanmei Yuan, Ranjing Tan, Bin Li, Gang Guo, Jinyong Zhang, Haiming Jing, Yi Qin, Zhuo Zhao, Quanming Zou, Chao Wu

**Affiliations:** ^1^ National Engineering Research Center of Immunological Products, Department of Microbiology and Biochemical Pharmacy, College of Pharmacy, Third Military Medical University, Chongqing, PR China; ^2^ Department of Dermatology, The Second Affiliated Hospital, Chongqing Medical University, Chongqing, PR China

**Keywords:** Helicobacter pylori, vaccine, th1 cell, th17 cell, immunodominant response

## Abstract

*Helicobacter pylori* has infected more than half of the world's population, causing gastritis, gastric ulcers, gastric mucosa-associated lymphoid tissue lymphoma and gastric cancer. The oral recombinant *Helicobacter pylori* vaccine currently used has made great progress in addressing this problem, however, its efficacy and longevity still need to be improved. Th1 and Th17 cells play essential roles in local protection against *Helicobacter pylori* in the stomach mucosa. Additionally, protective immunodominant antigens are the preferred for a vaccine. In this work, *Helicobacter pylori* whole cell lysate was separated into 30 groups based on molecular weight by molecular sieve chromatography. The group best promoting CD4 T cells proliferation was selected and evaluated by immunization. The detail proteins were then analyzed by LC-MS/MS and expressed in Escherichia coli. Eleven proteins were selected and the dominant ones were demonstrated. As a result, three protective immunodominant antigens, inosine 5'-monophosphate dehydrogenase, type II citrate synthase, and urease subunit beta, were selected from *Helicobacter pylori* whole cell. Two of them (inosine 5'-monophosphate dehydrogenase and type II citrate synthase) were newly identified, and one (urease subunit beta) was confirmed as previously reported. The mixture of the three antigens showed satisfactory protective efficiency, with significant lower H. pylori colonization level (*P* < 0.001) and stronger Th1 (*P* < 0.001) and Th17 (*P* < 0.001) responses than PBS control group. Thus, inosine 5'-monophosphate dehydrogenase, type II citrate synthase, and urease subunit beta are three protective antigens inducing dominant Th1 and Th17 responses to defend against *Helicobacter pylori* infection.

## INTRODUCTION

*Helicobacter pylori* (*H. pylori*) is a spiral-shaped, gram-negative bacterium that resides in gastric mucosa. It has infected more than half of the world’s population, causing gastritis, gastric ulcers, gastric mucosa-associated lymphoid tissue lymphoma (MALT) and gastric cancer [1]. The immunity responses induced by *H. pylori* are insufficient to clear *H. pylori*. Treatment with Bismuth triple therapy is efficacious but might cause antibiotic resistance, patient compliance and possible recurrence of *H. pylori* infection. Our oral recombinant *Helicobacter pylori* vaccine has made great progress in addressing this problem [2]. The fusion proteins consist of urease B subunit (gene derived from *H. pylori* 9803) and heat-labile enterotoxin B subunit (gene derived from E. *coli* H44815). In the phase 3 clinical trials, 4464 participants were involved. The oral vaccine was fully dissolved in distilled water and given on day 0, 14, and 28. Participants fasted for at least 2 h and were given 80 mL of buffer solution, containing 2.8 g sodium bicarbonate and 1.1 g sodium citrate, 2 min before the oral vaccination. The oral recombinant *H. pylori* vaccine showed an efficacy of 71.8% (95% CI 48.2–85.6) in the first year, but it decreased sharply to 55.0% (95% CI 0.9–81.0) in the second year. Its efficacy and longevity still need to be improved.

It has been proved by many researchers that Th1 and Th17 play essential roles in protecting against *H. pylori* in stomach mucosa [3–7]. Using the dominant epitope of CD4 T cells, HpaA_88-100_, we have demonstrated that the immunodominant CD4 T cell response to HpaA_88-100_ reduced the risk of severe gastric diseases in an HLA-DRB1^*^1501-restricted population [5]. Hitzler et al. analyzed the contribution of B cells, CD4 T cells, and dendritic cells to *H. pylori*-specific protection in immunized mice [6]. Their data showed that Th1 and Th17 but not humoral immunity protected against *H. pylori*. Similarly, DeLyria et al. also demonstrated that Th17 was protective in immunized mice against *H. pylori* [7]. However, the protective antigens inducing dominant Th1 and Th17 responses have never been systematically screened from *H. pylori*, which could be used to design a novel *H. pylori* vaccine based on them.

After *H. pylori* invades the gastric mucosa, *H. pylori* antigens are recognized and presented by antigen-presenting cells (APCs), such as dendritic cells (DCs) and macrophages [8]. Then, the APCs stimulate naïve CD4 T cells and induce antigen-specific responses in IFN-γ-secreting Th1 cells [9–11] and IL-17A-secreting Th17 cells [12, 13]. IL-17 promotes gastric epithelial cells, stromal cells, endothelial cells and lamina propria mononuclear cells (LPMC) that express the IL-17 receptor to release cytokines such as IL-1, IL-6, IL-8, and TNF-α, which then attract neutrophils to attack *H. pylori* [14].

We hypothesized it a better approach to select out the protective antigens inducing dominant Th1 and Th17 responses from *H. pylori* whole cell and design a novel vaccine. For this approach, each *H. pylori* protein was taken into consideration and assessed at the same time until the dominant ones were selected out. Meanwhile, protective efficacy and inflammation damage were measured in mice. As a result, three protective antigens, inosine 5′-monophosphate dehydrogenase (IMPDH), type Ⅱ citrate synthase (CS Ⅱ), and urease subunit beta (UreB), were shown to induce dominant Th1 and Th17 responses, with stronger *H. pylori* clearance and lower inflammation than PBS control. In addition, these three antigens represent new candidates for a novel vaccine in the coming years.

## RESULTS

### H. pylori whole cells were protective and elicited IFN-γ and IL-17A responses in mice stomach

**Figure 1:**
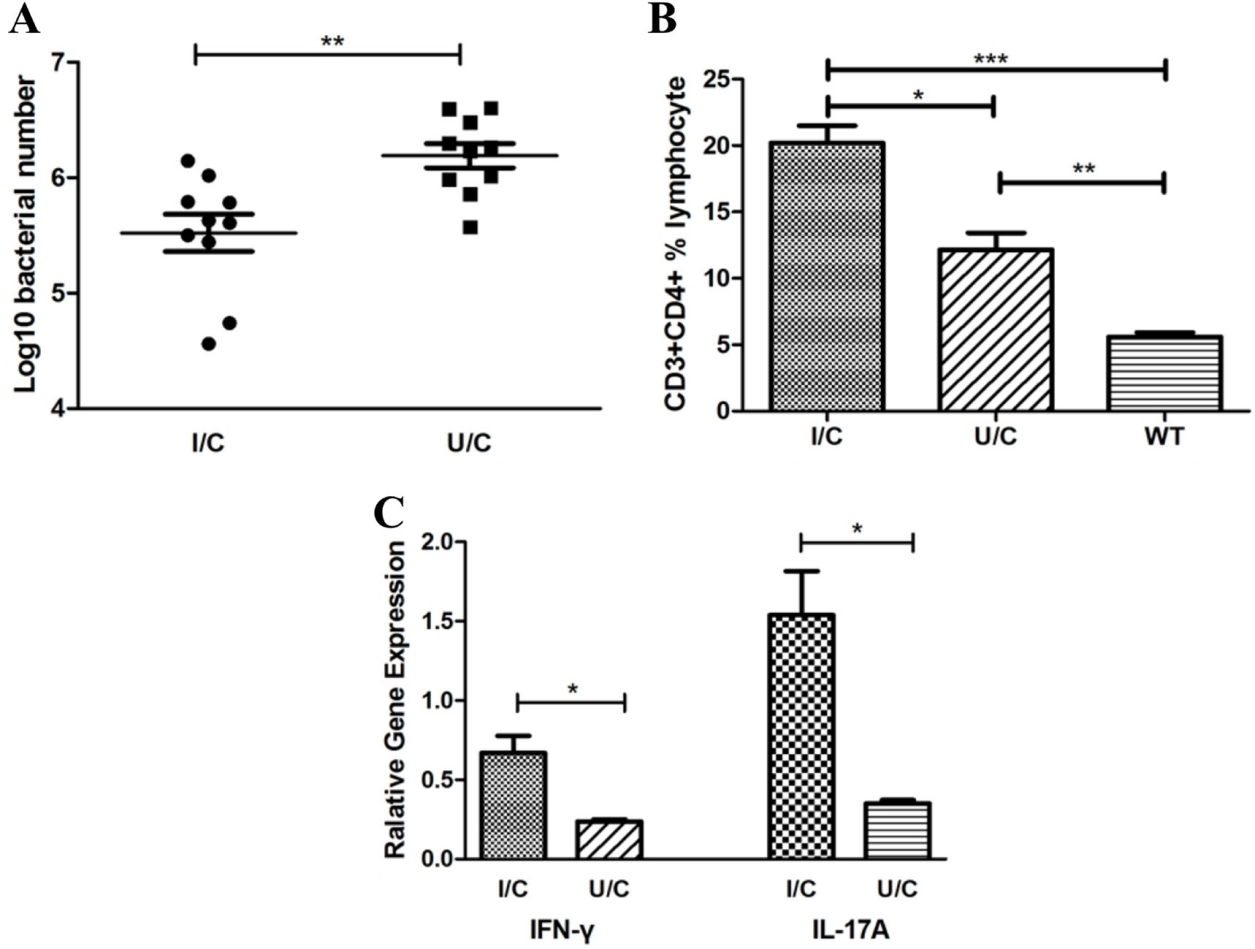
*H*. pylori whole cells (HWC) were protective and elicited IFN-γ and IL-17A responses in mouse stomachs Bacterial colonization and mRNA of cytokines in the stomachs were detected by quantitative real-time PCR. Single cell suspensions were isolated from the mouse stomachs and immunophenotyped by flow cytometry. (**A**) Bacterial colonization. Immunized and challenge group (I/C, log10 bacterial number, mean ± SD, 5.52 ± 0.51), unimmunized and challenge group (U/C, mean ± SD, 6.19 ± 0.33). *N* = 10, independent-sample *t*-test. (**B**) CD3 + CD4 + T cell population rate. I/C group (mean ± SD, 20.21% ± 2.22%), U/C group (mean ± SD, 12.16% ± 2.21%), and wild-type group (WT, mean ± SD, 5.59% ± 0.68%). *N* = 10, one-way ANOVA test with post-test Bonferoni. (**C**) IFN-γ and IL-17A responses in isolated gastric lymphocytes. *N* = 10, independent-sample *t*-test. ^*^P < 0.05, ^**^P < 0.01, ^***^P < 0.001.

To investigate whether *H. pylori* whole cells (HWC) were protective and induced CD4 T cell responses in the stomach, we immunized mice with inactivated *H. pylori* (group I/C, immunized/challenge) or PBS (group U/C, unimmunized/challenge) together with Freund’s adjuvant. Six- to eight-week-old BALB/c mice were immunized by subcutaneous injection with 100 μg HWC per mouse, emulsified in complete Freund’s adjuvant. Two weeks later, the immunization was boosted by 100 μg HWC combined with incomplete Freund’s adjuvant per mouse. After another two weeks, HWC without adjuvant were used for the last vaccination. The control group was immunized with the same protocol but using PBS instead of HWC. One week after the last boost, the mice were challenged with *H. pylori* strain B0 at 10^9^ CFU for each mouse each time, once a day for four days. Four weeks after the challenge, *H. pylori* colonization and immune responses were assessed. Our data showed that the bacterial colonization in group I/C (log10 bacterial number, mean ± standard deviation, 5.52 ± 0.51) was significantly lower than that in group U/C (mean ± SD, 6.19 ± 0.33, *P* < 0.01, Figure 1A). In addition, we found a much higher stomach CD3+CD4+ T cell population rate in group I/C (mean ± SD, I/C 20.21% ± 2.22%) than in group U/C (mean ± SD, 12.16% ± 2.21%, *P* < 0.05, Figure 1B). Meanwhile, group I/C exhibited significantly stronger IFN-γ and IL-17A responses based on the mRNA quantification of isolated gastric lymphocytes (*P* < 0.05, Figure 1C). Thus, IFN-γ and IL-17A responses were elicited and mice were protected from *H. pylori* challenge by *H. pylori* whole cell immunization.

### Dominant components promoted a higher level of CD4 T lymphocyte proliferation in the immunized/challenge group

**Figure 2:**
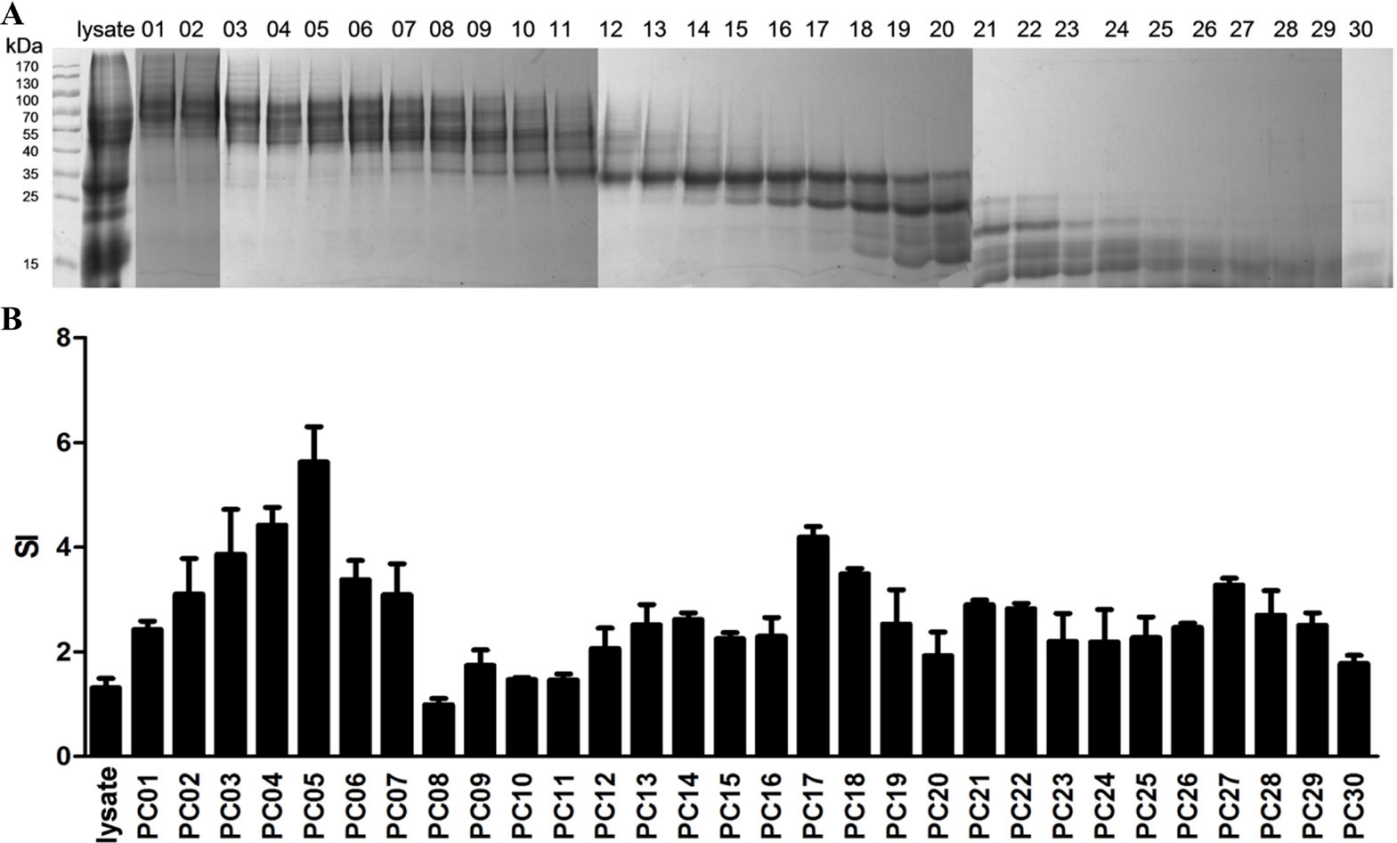
Selecting dominant mixed protein components that promoted CD4 T lymphocyte proliferation by ^3^H-TdR incorporation (**A**) *H. pylori* lysate was separated into 30 Mixed Protein Component groups (PC01 to PC30) based on their molecular weights by a molecular sieve chromatography method. All proteins groups were analyzed with SDS-PAGE. Five gels were used and each gel included a prestained protein ladder. The images of those five gels were combined together according to the prestained protein ladders. (**B**) CD4 T cell proliferation fingerprint of Immunized/Challenge group. APCs were cultured with *H. pylori* strain B0 whole cell lysate, PC01-30, or PBS in a 37°C incubator with 5% CO2 for 10 hours. Then spleen CD4 + T lymphocytes were sorted by immunomagnetic beads from the mice 4 weeks post-*H. pylori* challenge in the I/C group and stimulated with the APCs above for 96 hours. During the last 18 hours, 1 μCi [^3^H] thymidine (^3^H-TdR) was added. Counts per minute (cpm) were measured with a liquid scintillation counter, and the results of cell proliferation are expressed as stimulated indexes (SI), defined as the ratio of the cpm value of the experimental groups to the cpm value of the negative control group. *N* = 3.

CD4 T cells play an essential role in protecting humans against *H. pylori* infection. However, not every *H. pylori* antigen can stimulate a strong CD4 T cells response [15]. To determine the dominant *H. pylori* antigens that induce CD4 T cell responses, we first separated *H. pylori* lysate into 30 groups (PC01 to PC30) based on their molecular weights using a molecular sieve chromatography method (Figure 2A). Five gels were used and each gel included a prestained protein ladder. The images of those five gels were combined together according to the prestained protein ladders. Then, spleen CD4 T cells were sorted by immunomagnetic beads from the immunized/challenge group and stimulated with the PCs. As a result, CD4 T cell proliferation fingerprints showed a peak stimulated with PC05 and a subpeak stimulated with PC17 (Figure 2B). Therefore, PC05 and PC17 were selected and further evaluated.

### A dominant component (PC05) contributed more protection against H. pylori infection

**Figure 3:**
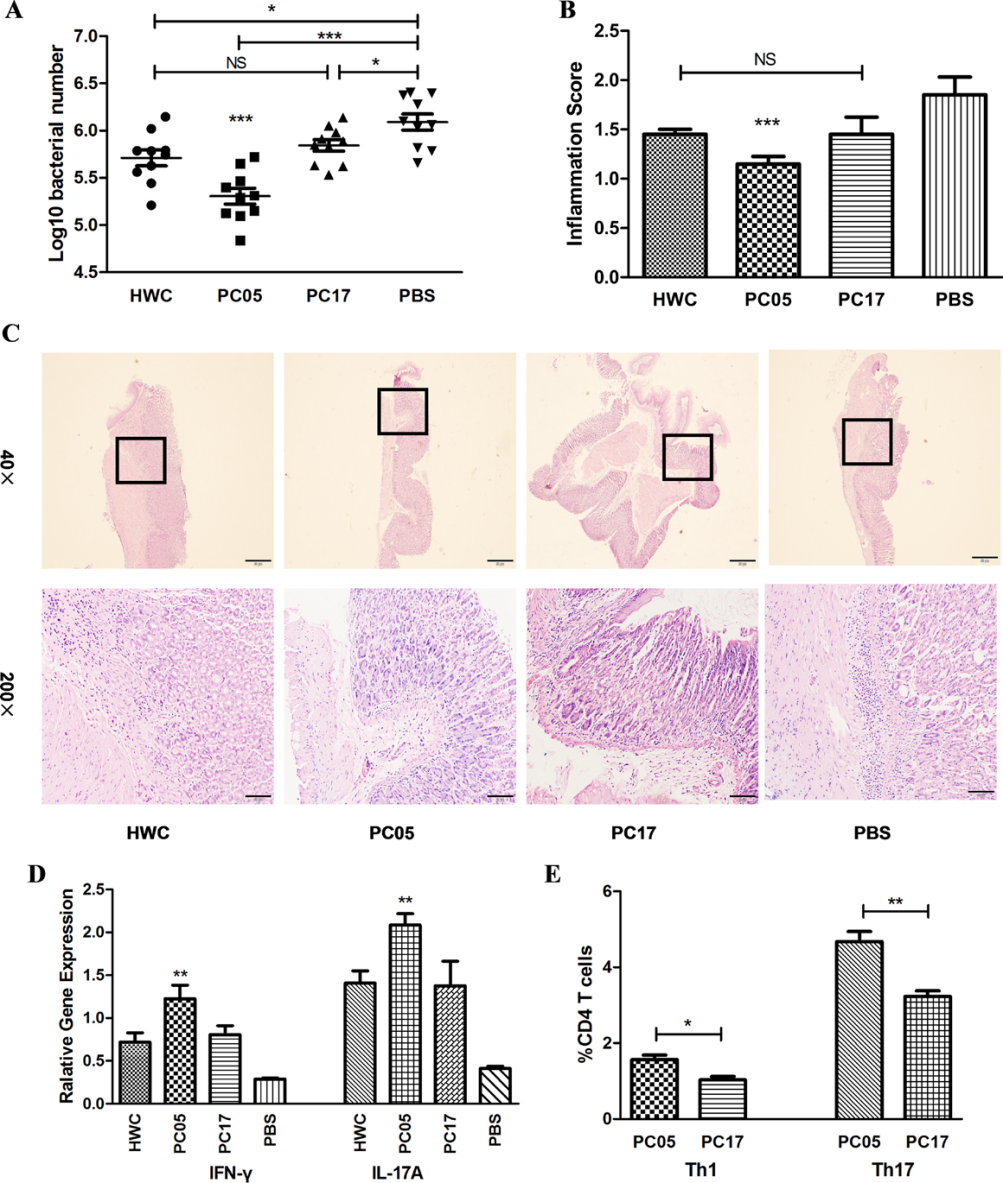
*H*. pylori whole cells (HWC), PC05, and PC17 vaccination and evaluation (**A**) Bacterial colonization in stomachs was detected by quantitative real-time PCR. *N* = 10, one-way ANOVA test with pos*t*-test Bonferoni. (**B**) Mouse stomach inflammation score. The histological evaluation was performed by two experienced pathologists in a blinded fashion. Gastric inflammation was graded on a 0 to 5 scale. *N* = 10, one-way ANOVA test with pos*t*-test Bonferoni. (**C**) Hematoxylin and eosin stained histological sections. Imaging at both low magnification (40×) and high magnification (200×). (**D**) INF-γ and IL-17A responses were evaluated in isolated stomach lymphocytes by quantitative real-time PCR at the mRNA level. *N* = 10, one-way ANOVA test with post-test Bonferoni. (**E**) PC05 induced dominant Th1 and Th17 responses in the spleen. Spleen lymphocytes were isolated from mice immunized with HWC and cultured with *H. pylori* lysate to obtain *H. pylori-*specific T cells. Then, the *H. pylori-*specific T cells were stimulated with PC05 or PC17. Th1 and Th17 responses were detected by flow cytometry. Data from 5 mice were analyzed with independent-sample *t*-tests. NS, not significant; ^*^P < 0.05, ^**^P < 0.01, ^***^P < 0.001.

Although the dominant antigen PC05 elicited the highest level of CD4 T cell proliferation *in vitro*, it was not clear whether it was the most protective mixed protein component *in vivo*. To answer this question, we immunized mice with HWC, PC05, PC17, or PBS as a control and challenged them afterwards. Regarding *H. pylori* colonization, the PC05 group showed the strongest clearance ability, with a significantly lower bacteria number in the stomach of the mice than that in the other three groups (*N* = 10, *P* < 0.001, one-way ANOVA test with post -test Bonferroni, Figure 3A). *H. pylori* colonization was not different between the HWC group and PC17 group. The *H. pylori* number in each of the three groups was lower than that in the PBS group. Meanwhile, the PC05 group exhibited less inflammation than the other three groups (*N* = 10, *P* < 0.001, one-way ANOVA test with pos*t*-test Bonferroni, Figure 3B–3C). The inflammation score also was not different between the PC17 group and HWC group. Additionally, we evaluated INF-γ and IL-17A responses with isolated stomach lymphocytes at the mRNA level. Our data showed that PC05 induced the strongest INF-γ and IL-17A responses among the four groups (*N* = 10, *P* < 0.01, one-way ANOVA test with pos*t*-test Bonferroni, Figure 3D). PC05 also induced dominant specific Th1 and Th17 responses in spleen. Spleen lymphocytes were isolated from mice immunized with HWC and then cultured with *H. pylori* lysate to obtain *H. pylori*-specific T cells. Then, the *H. pylori*-specific T cells were stimulated with PC05 or PC17. Th1 and Th17 responses were detected by flow cytometry. Similar to the results in the stomach, PC05 induced higher Th1 (*N* = 5, *P* < 0.05, independent-sample *t*-test) and Th17 (N = 5, *P* < 0.01, independent-sample *t*-test) responses than PC17 in the spleen (Figure 3E). Thus, it was clear that PC05 was more protective and was accompanied by stronger INF-γ and IL-17A responses.

### Identification of dominant antigens inducing stronger Th1 and Th17 responses from the PC05 component

**Figure 4:**
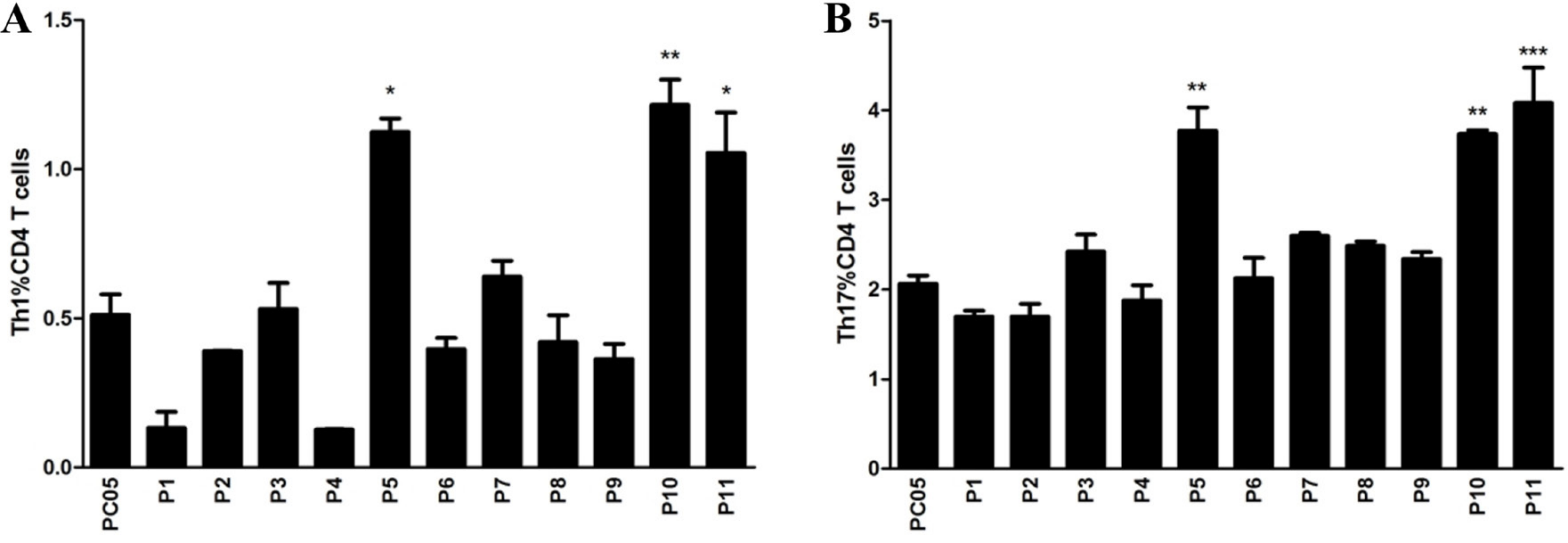
Identification of dominant antigens inducing stronger Th1 and Th17 responses from the PC05 component Spleen lymphocytes were isolated from mice immunized with PC05 and cultured with PC05 to obtain PC05-specific T cells. Then, the PC05-specific T cells were stimulated with P1 to P11. Th1 (**A**) and Th17 (**B**) responses were detected by flow cytometry. *N* = 3, one-way ANOVA test with post-test Bonferoni, ^*^P < 0.05, ^**^P < 0.01, ^***^P < 0.001.

PC05 induced dominant Th1 and Th17 responses not only in the stomach but also in the spleen, but the exact proteins in PC05 were unclear. To identify the dominant antigens in PC05 that induced strong Th1 and Th17 responses, PC05 was analyzed by LC-MS/MS, and 11 proteins were clarified, which were named P1 to P11 (Table 1). Then, we constructed expression vectors and expressed them in *Escherichia coli* (*E. coli*) to obtain the 11 proteins (purity > 88%, Supplementary Table 1). Endotoxin in the recombinant proteins was determined to be negative, as the values were lower than 10 EU/mg (Endotoxin Unit). Furthermore, spleen cells from PC05 immunized mice were stimulated with the PC05 component to expand specific lymphocytes *in vitro*. Then, PC05 specific lymphocytes were stimulated with P1 to P11 and Th1 and Th17 responses were detected by flow cytometry. The results showed that P5 (inosine 5′-monophosphate dehydrogenase, IMPDH), P10 (type Ⅱ citrate synthase, CS Ⅱ), and P11 (urease subunit beta, UreB) induced significantly higher Th1 and Th17 responses than the others did (N = 3, one-way ANOVA test with post -test Bonferroni, Figure 4A–4B).

**Table 1:** Eleven proteins were identified in PC05 by LC-MS/MS

NO.	Protein	Score	^#^AAs^*^	MW ^*^[kDa]	calc. pI^*^
P1	chaperonin GroEL	190.18	546	58.29	5.76
P2	elongation factor Tu	308.04	399	43.59	5.26
P3	GTP-binding protein TypA	20.42	599	66.68	5.44
P4	hydantoin utilization protein A	111.22	713	78.42	6.64
P5	inosine 5’-monophosphate dehydrogenase IMPDH	31.18	481	51.80	8.24
P6	leucyl aminopeptidase	42.78	496	54.53	7.09
P7	methyl-accepting chemotaxis protein McpA	61.65	433	48.38	6.25
P8	molecular chaperone DnaK	32.91	620	67.05	5.07
P9	protease DO	104.05	476	51.60	9.19
P10	type II citrate synthase CS II	29.06	426	48.32	7.88
P11	urease subunit beta UreB	483.10	569	61.55	6.01

^* #^AAs, amino acid number. MW, molecular weight. calc. pI, calculated isoelectric point.

### Three dominant antigens each had stronger protective roles against H. pylori challenge than the PC05 component

**Figure 5:**
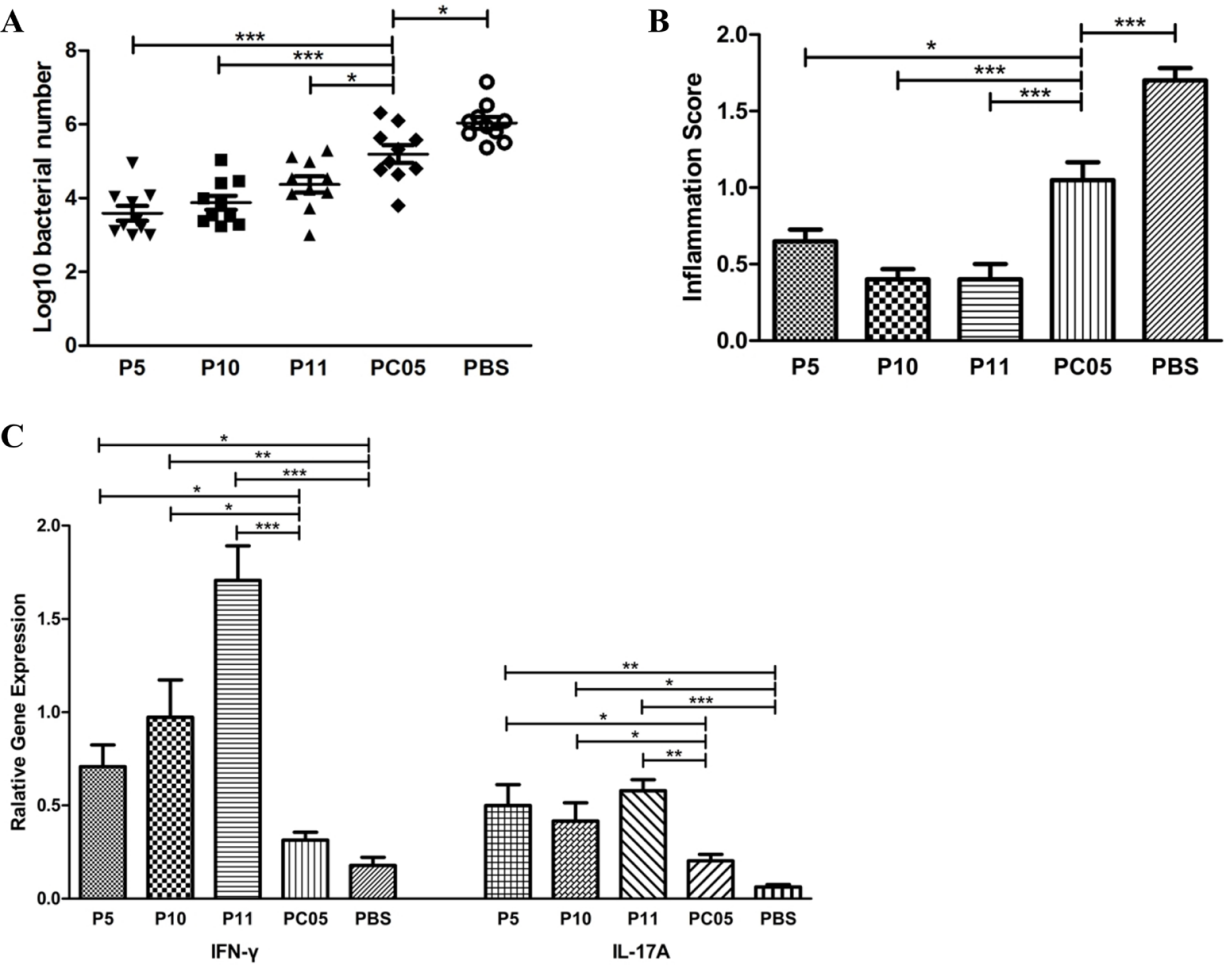
P5 (inosine 5′-monophosphate dehydrogenase, IMPDH), P10 (type II citrate synthase, CS II), and P11 (urease subunit beta, UreB) each had better protection against *H*. pylori challenge than the PC05 component Mice were immunized with P5, P10, P11, PC05, or PBS together with Freund’s adjuvant. (**A**) Bacterial colonization in stomachs was detected by quantitative real-time PCR. (**B**) Mouse stomach inflammation score. Hematoxylin and eosin stained histological sections were evaluated by two experienced pathologists in a blinded fashion. (**C**) INF-γ and IL-17A responses were evaluated in isolated stomach lymphocytes by quantitative real-time PCR at the mRNA level. *N* = 10, one-way ANOVA test with post-test Bonferoni, ^*^P < 0.05, ^**^P < 0.01, ^***^P < 0.001.

P5 (IMPDH), P10 (CS Ⅱ), and P11 (UreB) were shown to induce dominant Th1 and Th17 responses in PC05 component, but it was unclear whether each of them was protective. We immunized mice with the P5, P10, P11, or PC05 components or PBS as a control together with Freund’s adjuvant and then challenged with *H. pylori*. Regarding *H. pylori* colonization in the mouse stomach, the bacteria number in the P5 (*P* < 0.001), P10 (*P* < 0.001), and P11 (*P* < 0.05) groups was significantly lower than that in the PC05 group (*N* = 10, one-way ANOVA test with pos*t*-test Bonferroni, Figure 5A). Meanwhile, the bacteria number in the PBS group was higher than that in the PC05 group (*P* < 0.05). Regarding the inflammation score, all samples from the P5 (P < 0.05), P10 (P < 0.001), and P11 (P < 0.001) groups showed lower stomach inflammation score than those in the PC05 group (Figure 5B). In addition, stomach inflammation in the PBS group was much severer than that in the PC05 group (*P* < 0.001) (*N* = 10, one-way ANOVA test with pos*t*-test Bonferroni). Moreover, the responses of IFN-γ and IL-17A in the stomach were measured at the mRNA level. The data showed that P5, P10, and P11 all induced significant stronger IFN-γ and IL-17A responses than PC05 and PBS (*N* = 10, one-way ANOVA test with pos*t*-test Bonferroni, Figure 5C).

### Multi-subunit vaccine consisting of three dominant antigens had a better effect than a single-subunit vaccine

**Figure 6:**
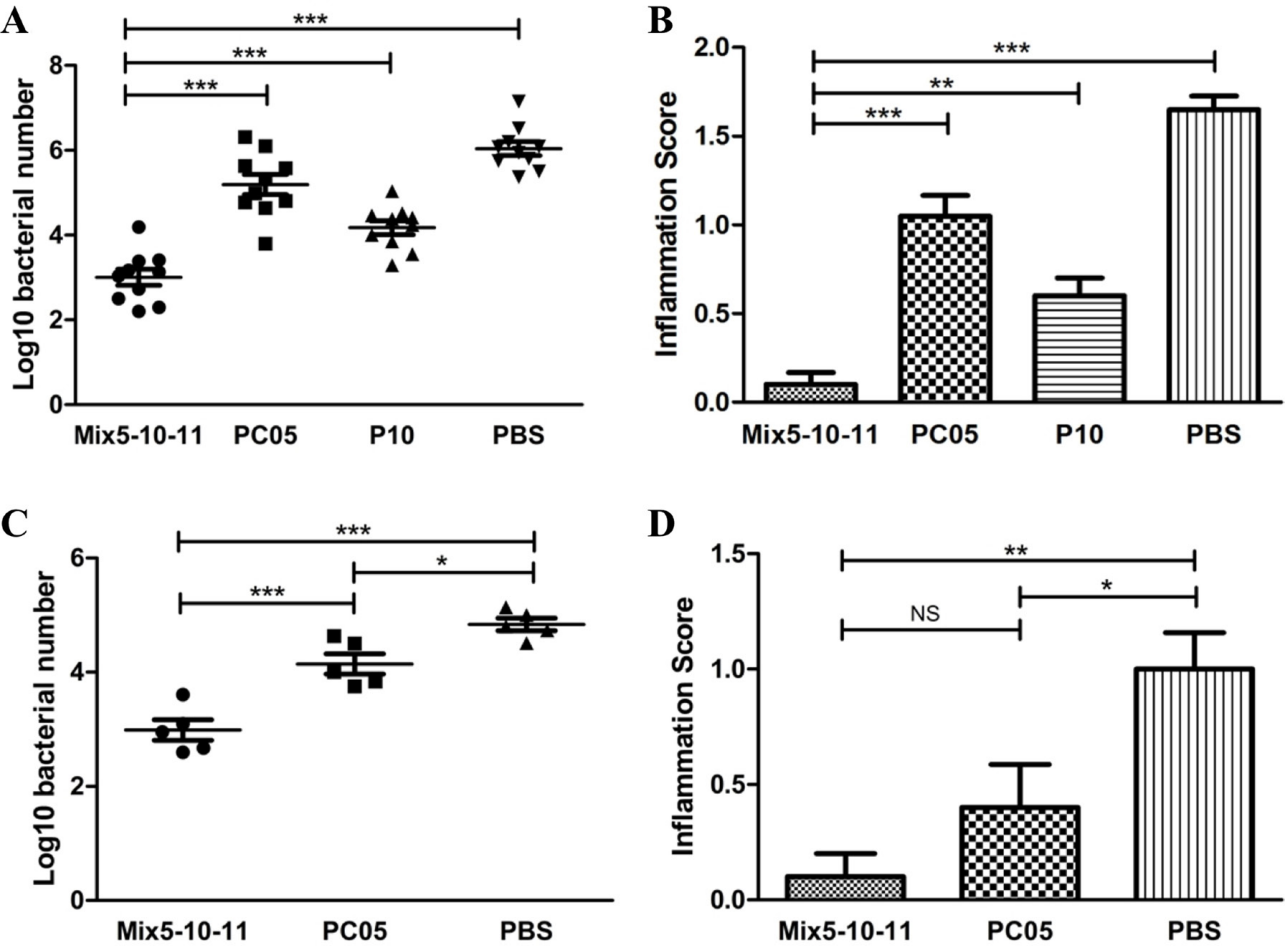
Multi-subunit vaccine consisting of three dominant antigens (Mix5-10-11) had a better efficiency than a single-subunit vaccine Mice were immunized with Mix5-10-11, PC05, P10, or PBS. Mix5-10-11 was more protective, resulting in lower *H. pylori* colonization (**A**) *N* = 10, and a lower inflammation score (**B**) *N* = 10. Mix5-10-11 or PC05-specific Th1 and Th17 splenic lymphocytes were cultured and sorted by immunomagnetic beads. Equal amounts of specific CD4 T cells or PBS were injected into mice. After *H. pylori* challenge, stomach bacteria number (**C**) *N* = 5, and inflammation score (**D**) *N* = 5 were evaluated. One-way ANOVA test with post-test Bonferoni. NS, not significant; ^*^P < 0.05, ^**^P < 0.01, ^***^P < 0.001.

Because P5 (IMPDH), P10 (CS Ⅱ), and P11 (UreB) were all good protective antigens, it was determined whether it would be better to immunize with a multi-subunit vaccine consisting of the three dominant antigens (mixture of P5, P10, and P11, Mix5-10–11). To clarify this issue, first, we immunized mice with Mix5-10-11, PC05, P10, PBS, P5, or P11 in the same way described above. The results showed that *H. pylori* colonization in Mix5-10-11 group was lower than that in PC05 (*P* < 0.001), P10 (*P* < 0.001), and PBS (*P* < 0.001) (*N* = 10, one-way ANOVA test with post - test Bonferroni, Figure 6A) groups as well as P5 and P11 groups (data not shown). Meanwhile, lower inflammation was induced in the Mix5-10-11 group than in the PC05 (*P* < 0.001), P10 (*P* < 0.01), and PBS (*P* < 0.001) (*N* = 10, one-way ANOVA test with pos*t*-test Bonferroni, Figure 6B) groups as well as the P5 and P11 groups (data not shown). Second, an adoptive cell experiment was carried out to evaluate the function of Mix5-10-11-specific CD4 T cells. Mix5-10-11- or PC05-specific splenic lymphocytes were cultured in the same way as described above. Then, equal amounts of CD4 T cells were sorted by immunomagnetic beads and injected into mice via the tail vein, at 2 × 10^6^ cells per mouse. The purity of CD4 T cells was 90.5% and 93.8%, respectively, in the Mix5-10-11 and PC05 groups. After the challenge, a lower stomach bacteria number was detected in the Mix5-10-11 group than in the PC05 group (*P* < 0.001) and PBS control group (*P* < 0.001, *N* = 5, one-way ANOVA test with pos*t*-test Bonferroni, Figure 6C). PC05-specific CD4 T cells were also protective compared with the PBS control group (*P* < 0.05). Moreover, lower inflammation was caused in both the Mix5-10-11 group (*P* < 0.01) and PC05 group (*P* < 0.05) than in the PBS group, whereas no significant difference was found between the Mix5-10-11 and PC05 groups (*N* = 5, one-way ANOVA test with pos*t*-test Bonferroni, Figure 6D).

### The Mix5-10-11 multi-subunit vaccine elicited stronger Th1 and Th17 responses

**Figure 7:**
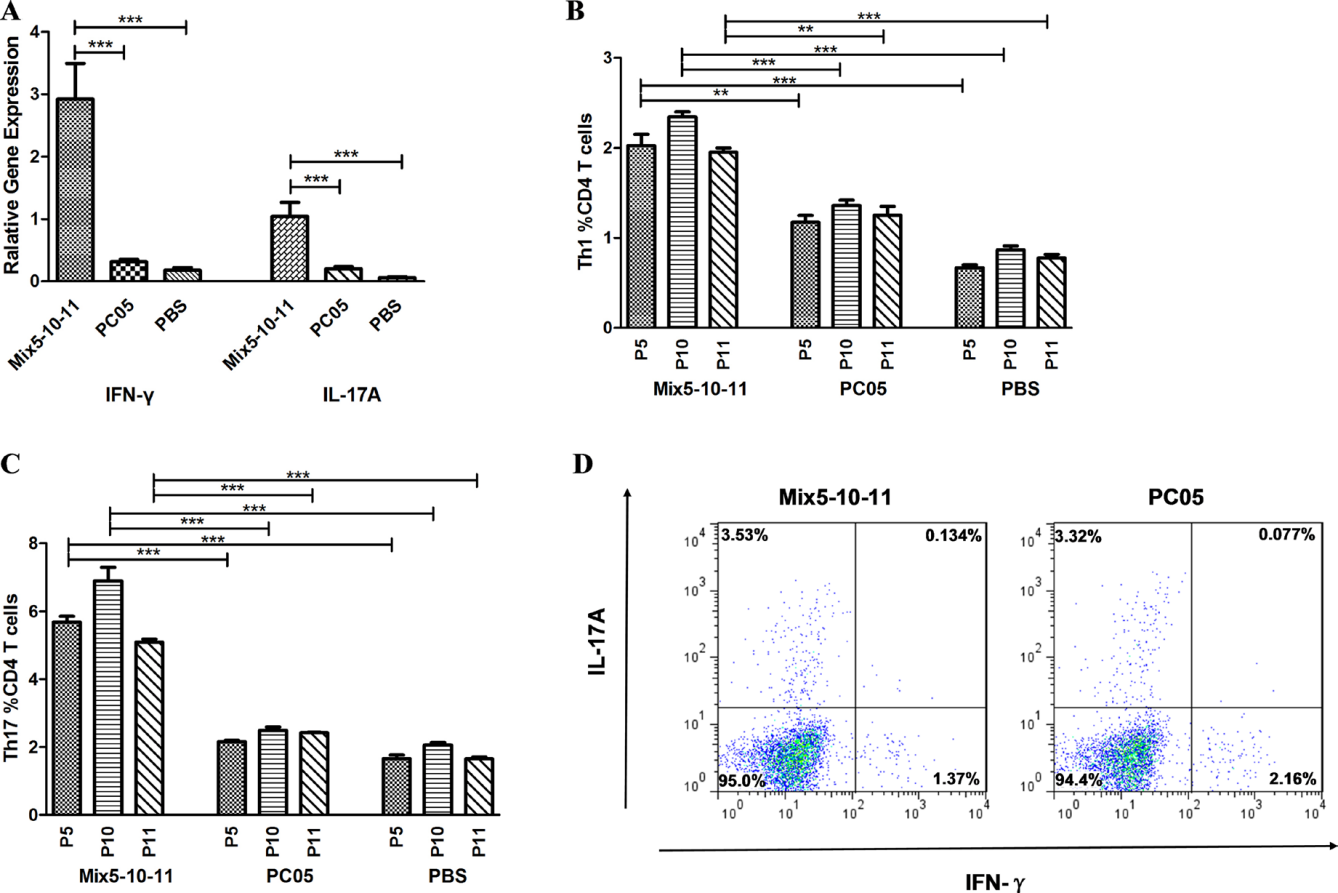
Mix5-10-11 multi-subunit vaccine elicited stronger Th1 and Th17 responses to protect against *H*. pylori infection Th1 and Th17 responses were evaluated in both multi-subunit vaccine Mix5-10-11 immunized mice and Mix5-10-11-specific CD4 T cell-adapted mice. In the Mix5-10-11, PC05, or PBS immunized/challenged groups, (**A**) mRNA levels of INF-γ and IL-17A were evaluated in isolated stomach lymphocytes with real-time PCR, *N* = 10. Meanwhile, spleen lymphocytes were isolated from the Mix5-10-11 or PC05 immunized mice and cultured with PC05 to obtain PC05-specific CD4 T cells. Then, the PC05-specific CD4 T cells from each group were stimulated with P5, P10, or P11. P5-, P10-, and P11-specific Th1 (**B**) and Th17 (**C**) responses were detected by flow cytometry, *N* = 5. (**D**) Th1 and Th17 responses were monitored in mice injected with Mix5-10-11 or PC05-specific CD4 T cells. Lymphocytes were isolated from the spleens of the Mix5-10-11 or PC05-specific CD4 T cell adapted mice and stimulated with Mix5-10-11 or PC05 respectively. Th1 and Th17 responses were monitored before *H. pylori* challenge to ensure that the transferred Mix5-10-11 or PC05-specific Th1 and Th17 cells were active. One-way ANOVA test with post-test Bonferoni. ^**^P < 0.01, ^***^P < 0.001.

Th1 and Th17 responses were evaluated in both multi-subunit vaccine Mix5-10-11 immunized mice and Mix5-10-11-specific CD4 T cell-adapted mice. In the Mix5-10-11 immunized mice, the stomach mRNA levels of IFN-γ (*P* < 0.001) and IL-17A (*P* < 0.001) were significantly higher than those in the PC05 group (*N* = 10, one-way ANOVA test with pos*t*-test Bonferroni, Figure 7A). Meanwhile, splenic lymphocytes were isolated from the Mix5-10-11 and PC05 groups and cultured with PC05 to obtain PC05-specific CD4 T cells. Then, the PC05-specific CD4 T cells from each group were stimulated with P5, P10, or P11. Th1 and Th17 responses were detected by flow cytometry. Similar with the mRNA level results, P5-, P10-, or P11-specific Th1 (*N* = 5, one-way ANOVA test with pos*t*-test Bonferroni, Figure 7B) and Th17 (*N* = 5, one-way ANOVA test with pos*t*-test Bonferroni, Figure 7C) responses were significantly stronger than those in the PC05 group. Lymphocytes were isolated from the spleens of the Mix5-10-11 and PC05-specific CD4 T cell adapted mice and stimulated with Mix5-10-11 or PC05, and Th1 and Th17 responses were monitored before *H. pylori* challenge to ensure that the transferred Mix5-10-11 or PC05-specific Th1 and Th17 cells were active (Figure 7D). The results indicated that the Mix5-10-11 multi-subunit vaccine induced dominant Th1 and Th17 responses to protect mice against *H. pylori* infection.

## DISCUSSION

It has been well demonstrated that CD4 T cells play essential roles in anti-*H. pylori* immunity. Previous studies have shown that it was MHC class Ⅱ-restricted antigens that protected against *H. pylori* infection [3]. We also previously demonstrated that CD4 T cell responses could reduce the risk of severe gastric diseases.[5] Similarly, our data showed that groups with stronger CD4 T cell responses had lower levels of *H. pylori* colonization in the stomach (Figure 1). Many studies on CD4 T cells drew our attention to Th1 and Th17 responses. Mattapallil et al. showed that *H. pylori* induced Th1 cells early during acute infection [16]. Additionally, the Th1 cell response was also demonstrated to be activated by gastric dendritic cells in humans.[10] Later, studies proved that the Th17 response was better able to protect against *H. pylori* infection when mice were immunized with inactive *H. pylori* [7, 17]. Moreover, immunization of mice with *H. pylori* whole cells elicited strong Th1 and Th17 responses in the mouse stomach according to our data (Figure 1). Thus, Th1 and Th17 responses seem to be essential, rather than those of other Th cells and humoral immunity [6, 18–20].

Antibody response has been paid much attention on in many vaccines development. Candidate protective antigens selected out accordingly show good efficiency in many researches [2, 21, 22]. Nevertheless, humoral immunity shows limited effects towards some specific pathogens or carcinoma. One reason for this might be apart from antibody response, T cell response is also necessary and usually much more essential [6, 7]. In *H. pylori* vaccine researches, our present oral recombinant *H. pylori* vaccine takes a big step to protect human against *H. pylori* infection. But, its efficacy and longevity still need to be improved. CD4 T cell responses should also be evaluated in *H. pylori* vaccine development.

T cells responses induced by *H. pylori* are insufficient to clear *H. pylori* in gastric mucosa. The bacterium persists and the inflammation continues for decades. Enhancing the protective ones through immunization can act as a strategy in developing *H. pylori* vaccine. Several *H. pylori* vaccines have successfully induced protection in animal models, such as vacuolating cytotoxin (VacA) [23], neutrophil-activating protein (NAP) [24], GroES and GroEL [25], but the efficacy in humans remains to be demonstrated.

The key step in the process is to select and evaluate candidate antigens for the *H. pylori* vaccine. Many antigens have been taken into consideration, including whole cell lysate, recombinant conserved antigens and strain-specific virulence factors. A variety of surface or secreted proteins have been demonstrated, such as VacA [23], NAP [24], CagA [26], UreB [27], GroES and GroEL [25]. What’s more, protective antigens HP0231 and HP0410 [28] was identified by a proteomic approach and multiparameter selection based on abundance, surface location, predicted T-cell epitopes, specificity and seroreactivity. However, *H. pylori* whole cells had never been screened systematically in any study. Because different from viruses, which have limited antigens, the hundreds of antigens in *H. pylori* whole cells hindered our efforts to select out the protective antigens. Finally, we achieved results with our strategy. In our study, we separated *H. pylori* lysate into 30 groups based on molecular weight using molecular sieve chromatography (Figure 2), and then, the specific proteins in the dominant mixed protein components were analyzed by LC-MS/MS (Figure 4). We successfully demonstrated that inosine 5′-monophosphate dehydrogenase (IMPDH), type Ⅱ citrate synthase (CS Ⅱ), and urease subunit beta (UreB) were three dominant antigens that induced Th1 and Th17 responses, and we then evaluated them in mice. Through our strategy, the published antigen UreB was confirmed to be a protective dominant antigen. The results also indicated that our strategy was effective and credible. Meanwhile, two novel protective dominant antigens, IMPDH and CS Ⅱ, were selected out and identified as candidate antigens for a novel *H. pylori* vaccine. Of course, besides the dominant mixed protein component PC05, some other mixed protein components also promoted CD4 T cells proliferation and may also contain protective candidate antigens for H. *pylori* vaccine design. It is meaningful to select out the dominant ones in those mixed protein components and then valued together with IMPDH, CS Ⅱ and UreB. Through that, we may get a better antigens combination to design a novel H. *pylori* vaccine. That’s what we are going to do the next step. All in all, our approach provides a new strategy for finding candidate antigens and designing vaccines for other pathogens.

With immunodominance, T cell responses consistently focus on one or several antigens, which are referred as immunodominant antigens. Immunization with immunodominant antigens can stimulate much stronger T cell responses. Thus, our results showed that it was better to use a dominant antigen, such as IMPDH, CS Ⅱ, or UreB, in a vaccine to elicit stronger Th1 and Th17 responses (Figure 5). In recent years, there has been a tendency in vaccine research to use multi-subunit vaccines. Compared with a single-subunit vaccine, a multi-subunit vaccine may show better efficacy and longevity with stronger immune responses [29]. Ferrero et al found the multi-subunit vaccine consisting of *H. pylori* GroES-like protein and UreB showing good protective efficiency in their research [25]. But further researches are still needed to design a successful multi-subunit H. pylori vaccine to be used in human beings [26, 30]. In our research, the multi-subunit vaccine (Mix5-10-11) was much more effective in mice than each of the P5 (IMPDH), P10 (CS Ⅱ), and P11 (UreB) single-subunit vaccines, especially that with UreB, which is the only antigen component in the present oral recombinant *H. pylori* vaccine [2]. Regarding *H. pylori* clearance, the Mix5-10–11 multi-subunit vaccine obviously had better results (Figure 6).

In our study, both the single-subunit vaccine (Figure 5A) and multi-subunit vaccine (Figure 6A) showed good efficacy, with low level of inflammation (Figure 5B, Figure 6B). However, effective immune protection has been reported to be associated with a strong inflammatory response in other studies [31]. which was inconsistent with our results. The reasons for that inconsistency may lie in the following. First, we used BALB/c background mice in our research. Previous research has demonstrated that *H. pylori* infection achieves high levels of colonization and causes less inflammation in BALB/c than C57BL/6 background mice [32]. Meanwhile, the inflammation is also related to the time post-*H. pylori* infection. In our study, we selected the time of 4 weeks after the last challenge to assess *H. pylori* colonization, histopathology, and immune responses. The inflammation may decline accordingly together with the clearance of bacteria. What’s more, the “inflammation score” here included not only infiltrate of inflammatory cells, but also epithelial hyperplasia, mucous cell metaplasia and so on. It was a comprehensive histological evaluation.

Here, Mix5-10-11 was shown to be protective only in a limited number of mice. Additional studies are needed to validate the protection of the multi-subunit vaccine using larger numbers of mice and Macaca rhesus monkeys as well as clinical trials together with an appropriate adjuvant. In addition, the candidate proteins can be reedited and recombined to obtain improved conservative and broader protection from *H. pylori*.

All in all, a new strategy to select protective candidate antigens was tried. That was based on Th1 and Th17 responses. And two new protective antigens IMPDH and CS II were demonstrated and the published antigen UreB was confirmed. It would further serve as a basement for epitope discovery and synthetic vaccine design in the coming years. What’s more, we provided a new strategy to be referred to select candidate antigens and design a vaccine for other pathogens.

## MATERIALS AND METHODS

### Bacteria and mice

The BALB/c mouse-adapted *H. pylori* strain B0 [33] was grown on brain-heart infusion agar with 10% rabbit blood at 37°C under microaerobic conditions. Two days later, the *H. pylori* strain was transferred from the plates to Brucella broth with 10% fetal bovine serum (FBS) to be actively cultured. The actively growing *H. pylori* strain was harvested for infection and *in vitro* experiments. To prepare inactivated *H. pylori* whole cells (HWC) for immunization, *H. pylori* cells were fixed in 0.3% formalin solution and incubated at 37°C for 6 hours and then washed three times with PBS. The 0.3% formalin fixed *H. pylori* was checked to confirm lack of viability by being grown on an agar plate. Six- to eight-week-old female BALB/c mice were infected with *H. pylori* strain B0 through intragastric administration with 10^9^ colony-forming units (CFU) for each mouse, once a day for four days. All mice were purchased from the Experimental Animal Center, Third Military Medical University and housed under specific pathogen-free (SPF) conditions. Animal maintenance and experimental procedures were carried out in accordance with the National Institutes of Health Guidelines for the Use of Experimental Animals and approved by the Medicine Animal Care Committee of the Third Military Medical University.

### Molecular sieve chromatography

First, to obtain *H. pylori* lysate, *H. pylori* strain B0 was dissolved in 8 mol/L urea with 10 mmol/L dithiothreitol(DTT) to 1 g/6 ml, stirring gently for 18 hours at 4°C. Second, the solution was centrifuged at 12,000 × *g*. The supernatant was then collected and passed through a 0.2-µm filter. Third, by molecular sieve chromatography, proteins with different molecular weight were separated into 30 groups, named Mixed Protein Component 01 (PC01) to PC30. A Superdex 200 10/300 GL column (GE Healthcare Life Sciences) was applied, and the flow rate was set to 0.5 ml/min [34]. All protein groups were analyzed with sodium dodecyl sulfate polyacrylamide gel electrophoresis (SDS-PAGE), and the concentration of each protein component was determined with a bicinchoninic acid (BCA) assay kit following the manufacturer’s instructions (Sigma-Aldrich Co. LLC. Louis, Missouri, USA).

### Vaccination and evaluation

Six- to eight-week-old female SPF BALB/c mice were immunized in the protocol we reported before [35]. Briefly, the mice were immunized by subcutaneous injection in the four limbs with 100 μg HWC, PC05, PC17, P5, P10, P11, or Mix5-10-11(P5:P10:P11 = 1:1:1) in 100 μl PBS, emulsified in 100 μl complete Freund’s adjuvant. As a result, 200 μl was used for each vaccine per mouse. Two weeks later, the immunization was boosted by 100 μg antigens combined with isometric incomplete Freund’s adjuvant per mouse. After another two weeks, antigens without adjuvant were used for the last vaccination. The control group was immunized with the same protocol but using PBS instead of antigens. One week after the last boost, the mice were infected with *H. pylori* strain B0 at 10^9^ CFU for each mouse each time once a day for four days. Four weeks after the infection, *H. pylori* colonization, histopathology, and immune responses were assessed.

### Isolating and immunophenotyping gastric lymphocytes

Gastric lymphocytes were isolated and immunophenotyped as previously published [36]. In brief, the stomachs were washed twice gently with PBS and cut into 2 equal pieces with a sharp scalpel along the greater curvature and lesser curvature. Each half of mice stomach was put into 10 ml Hank’s balanced salt solution (HBSS, without Ca, My) (Gibco) with 1 mM dithiothreitol (DTT), 1 mM ethylenediaminetetraacetate (EDTA), and 2% fetal calf serum (FCS) and incubated for 45 min at 37°C with gentle agitation. Then, the mixture was passed through sterile steel mesh to remove undigested tissues. The digested single-cell suspensions were harvested and washed twice in sterile PBS. Then, the digested single cells were marked by anti-mouse CD3 (FITC) and anti-mouse CD4 (APC) (eBioscience) and counted by flow cytometry.

### Histological evaluation

The longitudinal strips of stomach were formalin-fixed, embedded in paraffin, sectioned at 5 µm, and stained with hematoxylin and eosin. The histological evaluation was performed by two experienced pathologists in a blinded fashion. Gastric inflammation was graded on a 0 to 5 scale as previously reported [37]. Infiltrate of inflammatory cells, epithelial hyperplasia, mucous cell metaplasia and so on were included in the inflammation score evaluation.

### Quantitative real-time PCR

*H. pylori* colonization and immune responses in the stomachs were detected by quantitative real-time PCR. The bacterial genome was extracted from the mouse stomachs with a TIANamp Bacteria DNA Kit (TIANGEN, DP302) and dissolved in 200 μl deionized water. Then, 2 μl of *H. pylori* 16S rDNA was analyzed. For the quantitative real-time PCR, the target gene with a concentration gradient was set as standards, together with positive and negative controls. Two replicates were used for each sample. The detection result multiplied by 100 was regarded as the *H. pylori* colonization level in a mouse stomach, and the data are presented as log10 values (sense primer, 5′-TTTGTTAGAGAAGATAATGACGGTATCTAAC-3′, anti-sense primer, 5′-CATAGGATTTCACACCTGACTGACTATC-3′, *H. pylori* 16s probe, FAM-CGTGCCAGCAGCCGCGGT-TAMRA). Total RNA from the gastric single cells harvested above was extracted by TRIzol and reverse transcribed into cDNA with a PrimeScript TM 1st Strand cDNA Synthesis Kit (Takara). Then, the expression levels of interferon γ (IFN-γ) and interleukin 17A (IL-17A) were determined by quantitative real-time PCR with SYBR green staining (IFN-γsense primer, 5′-GATCCTTTGGACCCTCTGACTT-3′, IFN-γanti-sense primer, 5′- TGACTGTGCCGTGGCAGTAA-3′, IL-17A sense 5′-CTCCAGAAGGCCCTCAGACTAC-3′, IL-17A anti-sense 5′-GGGTCTTCATTGCGGTGG -3′).

### Antigen-specific splenic lymphocyte culture and detection

Spleen lymphocytes were mechanically isolated from HWC, PC05, or Mix5-10-11 immunized mice. Then, 1×10^7^ lymphocytes were cultured with 100 µg *H. pylori* lysate, PC05, or Mix5-10-11; 5 U/ml recombinant mouse interleukin-2 (rmIL-2) (PeproTech, Rocky Hill, NJ, USA); and 3 ml complete RPMI1640 medium in each well of a 12-well plate. Complete RPMI1640 medium consisted of RPMI 1640 (Gibco), 25 mmol/L hydroxyethyl piperazine ethanesulfonic acid (HEPES) pH 7.2, 100 U/ml penicillin, 100 μg/ml streptomycin, and 10% FBS. After being cultured for 5 days in a 37°C incubator with 5% CO_2_, dead cells were removed by Ficoll-Hypaque (TBDscience, Tianjin, China) gradient, and active lymphocytes were harvested and cultured in complete RPMI1640 medium with 20 U/ml rmIL-2 until analysis on the 12th day. During this period, half of the medium was changed when needed. Murine peritoneal macrophages from BALB/c mice were used as antigen-presenting cells (APCs). For this, 1×10^5^ APCs were placed in each well of a 96-well U-bottom plate with 200 µl of complete RPMI1640 medium. They were cultured with 50 µg/ml *H. pylori* lysate, protein components, protein, or PBS in a 37°C incubator with 5% CO_2_ for 10 hours. Then, the APCs were harvested and washed with complete RPMI1640 medium 3 times. Then, 1×10^5^ prepared APCs and 1×10^5^ lymphocytes were cocultured for 5 hours in each well of a 96-well U-bottom plate with 200 µl of complete RPMI1640 medium containing BD Golgistop (BD Biosciences, 554724). Then, the cells were stained with anti-mouse CD3 (FITC), anti-mouse CD4 (APC), anti-mouse IFN-γ (PE), and anti-mouse IL17A (PerCP-Cy5.5) and analyzed by flow cytometry.

### ^3^H-TdR incorporation

APCs were cultured with *H. pylori* strain B0 whole cell lysate, PC01-30, or PBS in a 37°C incubator with 5% CO_2_, and the final concentration of antigens in the culture was 50 µg/ml. Three replicates were used for each group. After 10 hours, spleen CD4+ T lymphocytes were sorted by immunomagnetic beads (MACS, Miltenyi Biotec) from the mice 4 weeks post-*H. pylori* challenge in HWC immunized/challenged group. Additionally, 1×10^5^ spleen CD4+ T lymphocytes were cocultured with 1×10^5^ APCs above in each well for 96 hours. During the last 18 hours, 1 μCi [^3^H] thymidine (^3^H-TdR) was added to each well. Then, counts per minute (cpm) were measured with a liquid scintillation counter, and the results of cell proliferation are expressed as stimulated indexes (SI), defined as the ratio of the cpm value of the experimental groups to the cpm value of the negative control group.

### LC-MS/MS analysis

The LC-MS/MS analysis was performed as we described in detail previously.[34] In brief, PC05 bands from the SDS-PAGE gel were digested with proteases and then detected with a maXis 4G UHR Q-TOF mass spectrometer. All data recorded were analyzed with Proteome Discoverer™ 1.4 software (Thermo Fisher Scientific, USA) and searched against the gene bank in the NCBI.fasta database (ftp://ftp.ncbi.nih.gov/). We thank the Biomedical Analysis Center, TMMU, for performing this analysis.

### Protein expression and purification

The target genes of the proteins P1-P6, P8-P10 were amplified by performing PCR from *H. pylori* DNA, with the primers shown in Supplementary Table 2. The genes of P7 and P11 were synthesized by Sangon Biotech (Shanghai, China). The pGEX-6P-1 expression plasmid was used as a vector plasmid. The target genes of the proteins were inserted into pGEX-6P-1 in the restriction sites shown in Supplementary Table 2. Then they were expressed in BL21(DE3) Competent *E. coli*. The target proteins were purified by Glutathione Sepharose 4 Fast Flow, and then, the GST-tag (26 kDa) was cut off with Prescission Protease (GE Healthcare Life Sciences). Endotoxin was further removed by ion exchange chromatography using an HiTrap Q HP column (5 ml). The endotoxin content was detected using the tachypleus amebocyte lysate test. Endotoxin in the recombinant proteins was determined to be negative, lower than 10 EU/mg (Endotoxin Unit). The purity of these proteins was determined by SDS-PAGE and high-performance liquid chromatography (HPLC). The concentration of these proteins was determined by the bicinchoninic acid (BCA) method.

### Adoptive transfer experiment

The adoptive cells experiment was carried out as described previously [35]. In short, *H. pylori* whole cells or Mix5-10-11-specific splenic lymphocytes were cultured, and the level of specific Th1 and Th17 responses was measured by flow cytometry. Then, CD4 T cells were sorted by immunomagnetic beads (Miltenyi Biotec) and transferred to mice via tail vein injection, at 2×10^6^ cells per mouse, in addition to measuring the purity of CD4 T cells. Lymphocytes were isolated from the spleen and stimulated with Mix5-10-11 or PC05, and Th1 and Th17 responses were monitored before *H. pylori* challenge by flow cytometry. After the above-described challenge, *H. pylori* colonization and inflammation were detected.

### Statistical analysis

Differences between two groups were analyzed by independent-sample *t*-tests. One-way ANOVA with pos*t*-test Bonferoni was used in comparisons of three or more groups. The statistical analysis was performed with SPSS16 software. A difference was regarded as significant when *P* < 0.05.

## SUPPLEMENTARY MATERIALS TABLES


