# Supplementary material for: Immunodominant antigens that induce Th1 and Th17 responses protect mice against Helicobacter pylori infection
Source: Oncotarget. 2018 Jan 3;9(15):12050–63. doi: 10.18632/oncotarget.23927 (PMC5844728; doi:10.18632/oncotarget.23927)
Supplement: Supplementary file 1 [file oncotarget-09-12050-s001.pdf]

Immunodominant antigens that induce Th1 and Th17 responses protect mice against *Helicobacter pylori* infection

SUPPLEMENTARY MATERIALS

Supplementary Table 1: Purity of proteins

NO.	Protein	MW [kDa]	Purity[%]	Endotoxin
P1	chaperonin GroEL	58.29	95.5	negative
P2	elongation factor Tu	43.59	91.0	negative
P3	GTP-binding protein TypA	66.68	88.1	negative
P4	hydantoin utilization protein A	78.42	90.6	negative
P5	inosine 5′-monophosphate dehydrogenase IMPDH	51.80	89.8	negative
P6	leucyl aminopeptidase	54.53	91.3	negative
P7	methyl-accepting chemotaxis protein McpA	48.38	94.3	negative
P8	molecular chaperone DnaK	67.05	94.8	negative
P9	protease DO	51.60	91.7	negative
P10	type II citrate synthase CS II	48.32	88.5	negative
P11	urease subunit beta UreB	61.55	98.2	negative

The purity of these proteins was determined by SDS-PAGE and high-performance liquid chromatography (HPLC). The endotoxin content was detected by the tachypleus amebocyte lysate test. Endotoxin in the recombinant protein was determined to be negative, lower than 10 EU/mg (Endotoxin Unit).

Supplementary Table 2: Primers used for amplifying the target genes of the proteins and corresponding restriction sites

NO.	Protein	Sense Primer	Anti-sense Primer	Restriction Sites	
P1	chaperonin GroEL	5′-CGCGGATCCATGGCA AAAGAAATCAAAT-3′	5′-CCGGAATTCTTAATCAGGCATTGCTGGG-3′	BamH I	EcoR I
P2	elongation factor Tu	5′-CGCGGATCCATGGCAAAAGAAAAG-3′	5′-CCGGAATTCTTATTCAATAATATTGCTC ACAAC-3′	BamH I	EcoR I
P3	GTP-binding protein TypA	5′-CGCGGATCCATGAAAAATA TTAGAAATATCG-3′	5′-CCGCTCGAGTTATTTTTTCGCCCTT-3′	BamH I	Xho I
P4	hydantoin utilization protein A	5′-CGCGGATCCATGAAAGACGCAAAAG-3′	5′-CCGGAATTCTTATTTAATTTC TTTCAAGTGGAAC-3′	BamH I	Xho I
P5	inosine 5′-monophosphate dehydrogenase IMPDH	5′-CCGGAATTCATGAGAATTT TACAAAGGGCT-3′	5′-CCGCTCGAGTTACCCATAATAATTAGGGGC-3′	EcoR I	Xho I
P6	leucyl aminopeptidase	5′-CGCGGATCCATGTTGAAAATCAAAT-3′	5′-CCGCTCGAGTTAAGCCTTTTTCAAAAATT-3′	BamH I	Xho I
P7	methyl-accepting chemotaxis protein McpA	Whole gene synthesis		BamH I	EcoR I
P8	molecular chaperone DnaK	5′-CGCGGATCCATGGGAAAAGTTATTG-3′	5′-CCGGAATTCTTACTCCACTTCCGCAT-3′	BamH I	EcoR I
P9	protease DO	5′-CGCGGATCCATGATGAAAAAAACC-3′	5′-CCGGAATTCTTATTTCACCAAAATGATCC-3′	BamH I	EcoR I
P10	type II citrate synthase CS II	5′-CGCGGATCCATGTCTGTTACTTTA-3′	5′-CCGGAATTCTTAATCCCCTACATAG-3′	BamH I	EcoR I
P11	urease subunit beta UreB	Whole gene synthesis		EcoR I	Xho I

The target genes of the proteins P1-P6, P8-P10 were amplified by performing PCR from H. pylori DNA, with the primers above. The genes of P7 and P11 were synthesized by Sangon Biotech (Shanghai, China). The pGEX-6P-1 expression plasmid was used as a vector plasmid. The target genes of the proteins were inserted into pGEX-6P-1 in the restriction sites shown above.
